# Exploring the neural basis of creativity: EEG analysis of power spectrum and functional connectivity during creative tasks in school-aged children

**DOI:** 10.3389/fncom.2025.1548620

**Published:** 2025-03-12

**Authors:** Gabriela Krumm, Vanessa Arán Filippetti, Magaly Catanzariti, Diego M. Mateos

**Affiliations:** ^1^Consejo Nacional de Investigaciones Científicas y Técnicas (CONICET), Ciudad Autónoma de Buenos Aires, Argentina; ^2^Centro Interdisciplinario de Investigaciones en Ciencias de la Salud y del Comportamiento (CIICSAC), Universidad Adventista del Plata, Libertador San Martín, Entre Ríos, Argentina; ^3^Instituto de Matemática Aplicada del Litoral (IMAL-CONICET-UNL), Santa Fe, Argentina; ^4^Facultad de Ciencia y Tecnología, Universidad Autónoma de Entre Ríos (UADER), Oro Verde, Entre Ríos, Argentina; ^5^Achucarro Basque Center for Neuroscience, Leioa, Bizkaia, Spain

**Keywords:** creativity, divergent thinking, TTCT, children, EEG, power spectrum, coherence, correlation

## Abstract

Creativity is a fundamental aspect of human cognition, particularly during childhood. Exploring creativity through electroencephalography (EEG) provides valuable insights into the brain mechanisms underlying this vital cognitive process. This study analyzed the power spectrum and functional connectivity of interhemispheric and intrahemispheric brain activity during creative tasks in 15 Argentine children aged 9 to 12, using a 14-channel EEG system. The Torrance test of creative thinking (TTCT) was used, incorporating one figural and one verbal task. EEG metrics included relative power spectral density (rPSD) across Delta, Theta, Alpha, Beta, and Gamma bands. Spearman's Rho correlations were calculated between frequency bands and performance on creativity tasks, followed by functional connectivity assessment through coherence analysis across the [1–50] Hz spectrum. The results revealed significant increases in rPSD across all frequency bands during creative tasks compared to rest, with no significant differences between figural and verbal tasks. Correlational analysis revealed positive associations between the Beta band and the innovative and adaptive factors of the figural task. In contrast, for the verbal task, both the Beta and Gamma bands were positively related to flexibility, while the Alpha band showed a negative relationship with fluency and originality. Coherence analysis showed enhanced intrahemispheric synchronization, particularly in frontotemporal and temporo-occipital regions, alongside reduced interhemispheric frontal coherence. These findings suggest that creativity in children involves a dynamic reorganization of brain activity, characterized by oscillatory activation and region-specific connectivity changes. Our study contributes to a deeper understanding of the brain mechanisms supporting creativity during child development.

## 1 Introduction

Creativity is characterized by the expression of new ideas, the ability to view things from different perspectives, and the capacity to combine unrelated concepts in novel ways (Benedek et al., 2012a,b). Creativity has been examined from multiple angles, including social, psychological, cognitive, and historical perspectives, resulting in a variety of theories (Amabile, 1983; Csikszentmihalyi, 1988; Guilford, 1956; Mednick, 1962; Simonton, 1988; Sternberg and Lubart, 1993). However, the brain mechanisms underlying creative thinking, particularly in children, remain not fully understood.

Creativity arises from basics mental processes (Boden, 1998), linking it, to cognitive science and neuroscience. Theories of creativity must align with the current understanding of brain function (Pfenninger and Shubik, 2001). Recent research indicates that creativity is not a single, unified faculty, but rather evolves from the dynamic interaction among various distributed neural networks (Dietrich, 2024; Pearl, 2024). This creative process is not isolated; it builds upon prior knowledge and is enriched through the combination of different perspectives. Current neuroscientific evidence indicates that creativity requires both the activation of networks associated with the default mode during spontaneous idea generation and the involvement of central executive networks during the elaboration and refinement of these ideas (Pearl, 2024).

### 1.1 EEG and creativity

Research employing electroencephalography (EEG) has extensively studied brain activity during creative processes, highlighting variations in neuronal activity patterns across diverse creative tasks, including those that assess remote associations and artistic expressions such as creative storytelling, metaphors, humor, paintings, and melodies (Arden et al., 2010; Dietrich and Kanso, 2010; Bazanova and Aftanas, 2008; Danko et al., 2009; Fink and Benedek, 2014; Fink et al., 2009; Grabner et al., 2007; Razumnikova et al., 2009; Pidgeon et al., 2016; Rominger et al., 2019; Stevens Jr and Zabelina, 2019; Sun and Zhou, 2024; Volf and Razumnikova, 1999). Research on the power spectrum in young adults indicates that significant changes occur during creative tasks (Rominger et al., 2019; Volf and Razumnikova, 1999). These changes are particularly evident in the alpha band across the frontal, parietal-occipital, and right hemispheric regions (Fink et al., 2009; Grabner et al., 2007; Jauk et al., 2012; Rominger et al., 2019). This activity has been associated with internal attention processes (Benedek, 2018) and the inhibition of irrelevant stimuli, decreasing when attention is focused outward (Benedek et al., 2011; Stevens Jr and Zabelina, 2020). Consistently, in their study on EEG and creativity, Ahad et al. (2023) analyzed brain patterns during creative ideation, finding a marked decrease in alpha power in the parieto-occipital region (O1/2, P7/8). This pattern could be explained by the differential activation of specific brain regions: while alpha synchronization in the frontal region indicates high demands for internal processing, desynchronization in the posterior areas (parieto-occipital) reflects a greater demand on the visual system during creative processing. In this regard, alpha synchronization has been correlated with divergent thinking, as opposed to convergent thinking, especially in fronto-parietal areas. Alpha synchronization is interpreted as top-down processing and internal attention, while alpha desynchronization, more prominent during convergent tasks, is related to remote associations and the consolidation of semantic memory. These patterns suggest that modulation of the alpha band is linked to the specific processing demands that each creative task requires, rather than to creative cognition itself (Eymann et al., 2024). Earlier brain imaging studies have reported that different frequency bands, such as delta, theta, beta, and gamma, also show distinctive patterns during creative tasks (see e.g., Boot et al., 2017; Wokke et al., 2019). Specifically, changes in beta and gamma bands in the temporal and central brain regions, along with a decrease in theta in parieto-occipital areas, have been associated with the creative process (Danko et al., 2009; Pidgeon et al., 2016; Shemyakina et al., 2007). Studies in this line, have documented changes in alpha and gamma oscillations associated with creative idea generation and problem-solving (Jauk et al., 2012). Jung-Beeman et al. (2004) identified specific changes in theta and beta oscillations during creative insight phases. Regarding the theta band, an increase in fronto-occipital functional connectivity has been observed in individuals with high creativity (Wokke et al., 2019; Cavanagh and Frank, 2014). Razumnikova et al. (2009) found that theta coherence increases during visual tasks and decreases during verbal tasks, while beta activity increases during visual tasks compared to the baseline. Additionally, Volf and Razumnikova (1999) documented that high levels of creativity are associated with theta and beta activity in frontal-occipital and lateral regions, while Bhattacharya and Petsche (2005) observed notable changes in neural synchronization patterns, with emphasis on the theta band during creative problem-solving. More recently, Bartoli et al. (2024) examined creativity's neurophysiological mechanisms using direct brain recordings (EEG) during divergent thinking tasks. Their findings revealed specific patterns in the Default Mode Network (DMN): an increase in gamma waves (30–70 Hz) and a decrease in theta waves (4–8 Hz), especially in lateral temporal regions during the initial phase of creative processing. As the authors note: “DMN activity was characterized by a stronger increase in gamma band power coupled with lower theta band power” (p. 3409), providing crucial evidence on the brain mechanisms underlying the generation of original ideas.

EEG studies have revealed specific activation patterns regarding the key brain regions involved in the creative process. For example, Beaty et al. (2015) found increased activation in the frontal areas, particularly in the dorsolateral prefrontal cortex (DLPFC) during creative tasks, while Fink et al. (2009) documented significant synchronization patterns between frontal and temporal areas. Rominger et al. (2022) highlighted that brain activation patterns during the generation and evaluation of creative ideas vary by brain region, reflecting different aspects of the creative process. Increases in parietal and occipital areas would be linked to internal attention and inhibition of external information, while changes in temporal regions would be related to memory and associations. A decrease in alpha power in the parietal and occipital areas is associated with heightened sensory processing and convergent thinking. Conversely, an increase in alpha power in these regions is linked to greater internal attention and working memory, particularly in individuals with lower levels of metacognitive monitoring.

The heterogeneity in EEG findings can be explained in a more fundamental way. In this regard, Dietrich (2024) suggests that conceptualizing creativity as a unitary brain faculty is problematic. The neurocognitive mechanisms of creativity are diverse and depend on the specific type of creative process involved. This suggests that there is no single neural pattern that can define all forms of creative activity. Different creative tasks show unique activation patterns in EEG readings, as each type of creativity activates different neural networks. This view of distributed processes is supported by recent research on brain networks and creativity (Pearl, 2024). Research involving musicians has highlighted two key networks during improvisation: the default mode network (DMN), which is engaged in the medial prefrontal cortex during creative improvisation, and the central executive network (CEN), activated during a repetitive musical performance. Neuroimaging studies indicate that during improvisation, the medial prefrontal cortex becomes activated, while the dorsolateral and lateral orbital prefrontal regions are deactivated. This suggests that creativity arises from the interaction of different neural networks. A relaxed mental state can enhance creativity, while heightened executive control may inhibit it (Pearl, 2024).

Current studies highlight the importance of clearly defining creativity and distinguishing it from other traditional mental abilities, such as intelligence (Fink and Benedek, 2014). In this regard, EEG represents a suitable tool for studying creativity under optimal conditions (Fink and Benedek, 2014). Additionally, when examining the specific neurocognitive processes linked to creativity, it is essential to use widely recognized tasks with strong psychometric properties, such as the Torrance Test of Creative Thinking (1990). Particularly, the use of both verbal and figural tasks from the TTCT in children is highly relevant, as these tasks capture different aspects of divergent thinking during this crucial stage of development. The figural tasks evaluate visuospatial creative abilities (Torrance et al., 1992), while the verbal tasks focus on creative linguistic expression (Torrance, 1990). This complementary approach could provide a more comprehensive understanding of children's creativity and its cognitive foundations. To our knowledge, no research has examined brain connectivity/dysconnectivity during TTCT performance in typically developing children. Investigating these aspects of brain function is crucial, as it allows us to quantify brain regions that exhibit increased or decreased information exchange during cognitive tasks compared to a baseline state. This understanding deepens our insight into the brain-behavior relationships. Therefore, this study aims to analyze the power spectrum, the correlation between the EEG-based parameters and the behavioral results, and the functional inter- and intra-hemispheric brain connectivity and dysconnectivity during the TTCT performance test in school-aged children.

## 2 Materials and methods

### 2.1 Participants

The sample consisted of 15 Argentine children aged 9–12 years (M = 10.33; SD = 1.11) from a middle socioeconomic level. Inclusion criteria included: absence of neurological or psychiatric history, normal or corrected sensory abilities, regular school attendance, and no grade repetition. IQ, levels of inattention and hyperactivity-impulsivity, and parental educational level on a five-level scale from primary to postgraduate studies were evaluated. The assessments were conducted individually in two sessions using the Emotiv Epoc+ EEG recording device, which was introduced to interested parents beforehand. Informed consent was obtained from parents or legal guardians, and approval was granted by the Ethics Committee of FCS-UAP (Resolution 5.7/2019). We present a table detailing the sociodemographic characteristics of the sample in the Supplementary Table S1.

### 2.2 Data acquisition

**Figure 1:**
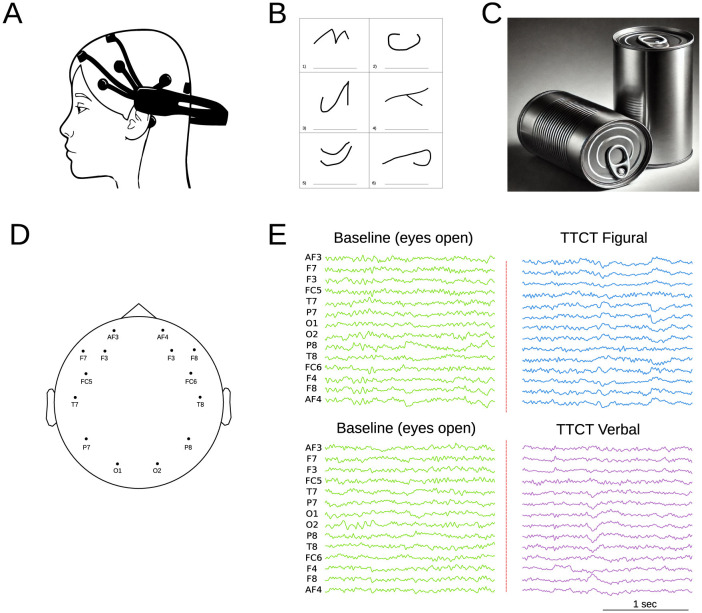
**(A)** Schematic representation of the Emotiv Epoch system. **(B)** Example of the TTCT Figural Test (Task 2), which involves completing incomplete figures and assigning titles within a 10-min timeframe. **(C)** Example of the TTCT Verbal Form B (Task 5), where participants list unusual uses for a specific object (cans in this case) over 10 min. **(D)** EEG electrode topographic brain distribution. **(E)** Example of EEG signal recording, starting with an initial baseline measurement (open eyes) followed by recordings during the different test tasks.

The EEG data were collected using the Emotiv Epoc+ device (Emotiv, 2021) (Figure 1A), which features 14 electrodes positioned according to the international 10–20 system (AF3, F3, F7, FC5, T7, P7, O1, O2, P8, T8, FC6, F8, F4, and AF4) and two reference electrodes (see Figure 1D). The acquisition sampling frequency was 128 Hz. Previous studies have demonstrated that the Emotiv Epoc headset reliably captures high-resolution patterns of brain activity (Bobrov et al., 2011) and exhibits strong test-retest reliability (Amjad et al., 2019). Data acquisition was conducted separately for each test. The process consisted of two stages: an initial stage where data were recorded 5 min from the children in a resting state with their eyes open (control condition), followed immediately by recordings taken while they performed the assigned tasks (see Figure 1E).

### 2.3 Cognitive tasks

#### The Raven's progressive matrices test

It assesses general intellectual ability (fluid intelligence). The assessment scale used varied depending on the age of the children in the sample (Raven and Raven, 2008). For children between 5 and 11 years old, the Color Scale (RCPM) was utilized, which consists of three series. For 12-year-old children, the General Scale (RPM) was employed, which includes five series.

#### The Swanson, Nolan, and Pelham rating scale–fourth version

This scale is designed to assess hyperactivity, impulsivity, and attention deficits in children. It features two versions: one for parents and one for teachers. Each symptom is rated on a scale from 0 to 3, with nine items focusing on attention and nine addressing hyperactivity and impulsivity. The total score can range from 0 to 27. In Argentina, the teacher version of this scale has been utilized as a tool for identifying Attention Deficit Hyperactivity Disorder (ADHD) in children aged 4 to 14 (Grañana et al., 2011).

#### The TTCT figural form A, task 2

This task (Torrance et al., 1992) requires completing incomplete figures and assigning titles to them in a time frame of 10 min. It measures creativity through fluency, originality, elaboration, resistance to premature closure, and abstract title (Figure 1B). These dimensions produce two correlated factors: Innovative (fluency and originality) and adaptative (elaboration, resistance to premature closure, and abstraction of titles). Confirmatory Factor Analysis (CFA) with Argentine children confirmed this bifactorial structure, showing invariance by gender (Krumm et al., 2016b).

#### The TTCT verbal form B, task 5

This task (Torrance, 1990) requires listing unusual uses for a specific product for 10 min (Figure 1C). The task evaluates three dimensions of creative thinking: fluency (total number of relevant responses), flexibility (diversity of categories), and originality (novelty of ideas), excluding non-creative responses. CFA with young Argentine adults showed a better fit for the six-factor model, where each activity represents a correlated factor that evaluates the three dimensions (Krumm et al., 2016a).

### 2.4 Data analysis

#### 2.4.1 Preprocessing

The first step involved preprocessing the EEG signals. A bandpass filter was applied, which limits the frequency range to [0.5–50] Hz. Channels with poor signal quality were manually identified and replaced using an interpolation algorithm. The signal was then segmented into 5-second epochs. A visual inspection of the data was conducted to identify and remove epochs affected by movement artifacts or other acquisition issues. One participant was excluded from the analysis due to excessive noise in the EEG recording. The epochs from both baselines (figural and verbal tasks) were normalized using z-score approach. On average, each participant have 20 ± 3 claen epoch in baseline and 52 ± 7 in each the test (Figure 1E). Then independent component analysis (ICA) was performed to isolate and remove components associated with myographic, cardiac, and ocular artifacts. After completing the preprocessing steps, the remaining epochs were categorized into two conditions: baseline (BL) and task performance (figure or verbal task). All analyses were conducted using the open-source MNI Python Package (Gramfort et al., 2013).

#### 2.4.2 Power spectrum analysis

The first analysis focused on the relative power spectrum density (rPSD), a normalized metric that quantifies the contribution of a specific frequency band relative to the total power across all frequency bands. The frequency bands analyzed included Delta [1–4] Hz, Theta [4–8] Hz, Alpha [8–12] Hz, Beta [12–30] Hz, and Gamma [30–50] Hz. Spectral analysis was performed with a resolution of 0.25 Hz across the frequency range of 0.5 to 50 Hz, using the Welch method implemented in the SciPy signal package in Python. Segments were 1,000 points long, and the butterworth filter was applied with third-order configuration.

#### 2.4.3 Correlation analysis

Spearman's Rho correlations were calculated between the Figural and Verbal factors of the TTCT and the frequency bands (Delta, Theta, Alpha, Beta, and Gamma). This non-parametric test was chosen due to the small sample size and because the Shapiro-Wilk test confirmed a non-normal distribution of the data, especially in the Gamma band (*p* < 0.05).

#### 2.4.4 Functional connectivity: coherence

Coherence analysis was used to evaluate functional connectivity between EEG signals across different channels. Coherence measures the degree of linear synchronization between two signals based on their phase and amplitude relationships (Stoica and Moses, 2005). Coherence values were computed using Welch's method. The analysis was performed in Python using the function scipy.signal.coherence, with a Hann window of 800 ms and 50% overlap. Coherence was analyzed as a function of frequency within the range of [2 − 50] Hz for all channel pairs. The mean coherence was calculated across all epochs corresponding to the same participant and experimental condition. Subsequently, a statistical analysis was conducted to compare coherence values across the different groups: baseline, verbal test, and figure test.

## 3 Results

### 3.1 Relative power spectrum density analysis

**Figure 2:**
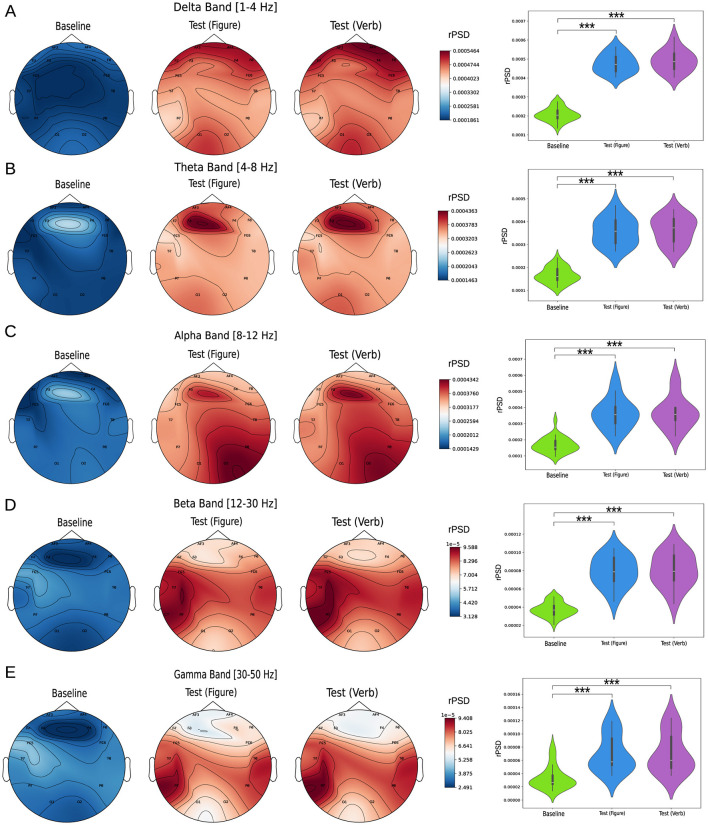
Relative power spectrum density analysis (rPSD). The left column shows topoplots of rPSD values across the scalp for the following frequency bands: **(A)** Delta [1–4 Hz], **(B)** Theta [4–8] Hz, **(C)** Alpha [8–12] Hz, **(D)** Beta [12–30] Hz, and **(E)** Gamma [30–50] Hz. The right column compares rPSD values between groups, with each violin plot representing the average value across all measured channels. Statistical comparisons between conditions were performed using the Kruskal-Wallis test, followed by Dunn's multiple comparisons test to identify significant differences (^***^p < 0.0001).

The relative power spectrum density (rPSD) was analyzed across the Delta, Theta, Alpha, Beta, and Gamma bands for all EEG channels, comparing three conditions: baseline, figure test, and verbal test. Figure 2 left column illustrates the rPSD values distributed across the brain topology, along with the average values across all channels (Figure 2 right column). For all frequency bands, we observed a consistent pattern: rPSD values during the baseline state were significantly lower than those during the cognitive tasks. This difference was statistically significant across all measured channels (*p*-values for each channel are presented in Supplementary Figure S1). These findings are even more pronounced when averaging across all channels. Additionally, no significant differences in rPSD were observed between the two tests (Figure and Verbal) for any of the frequency bands analyzed.

### 3.2 Correlation analysis

**Figure 3:**
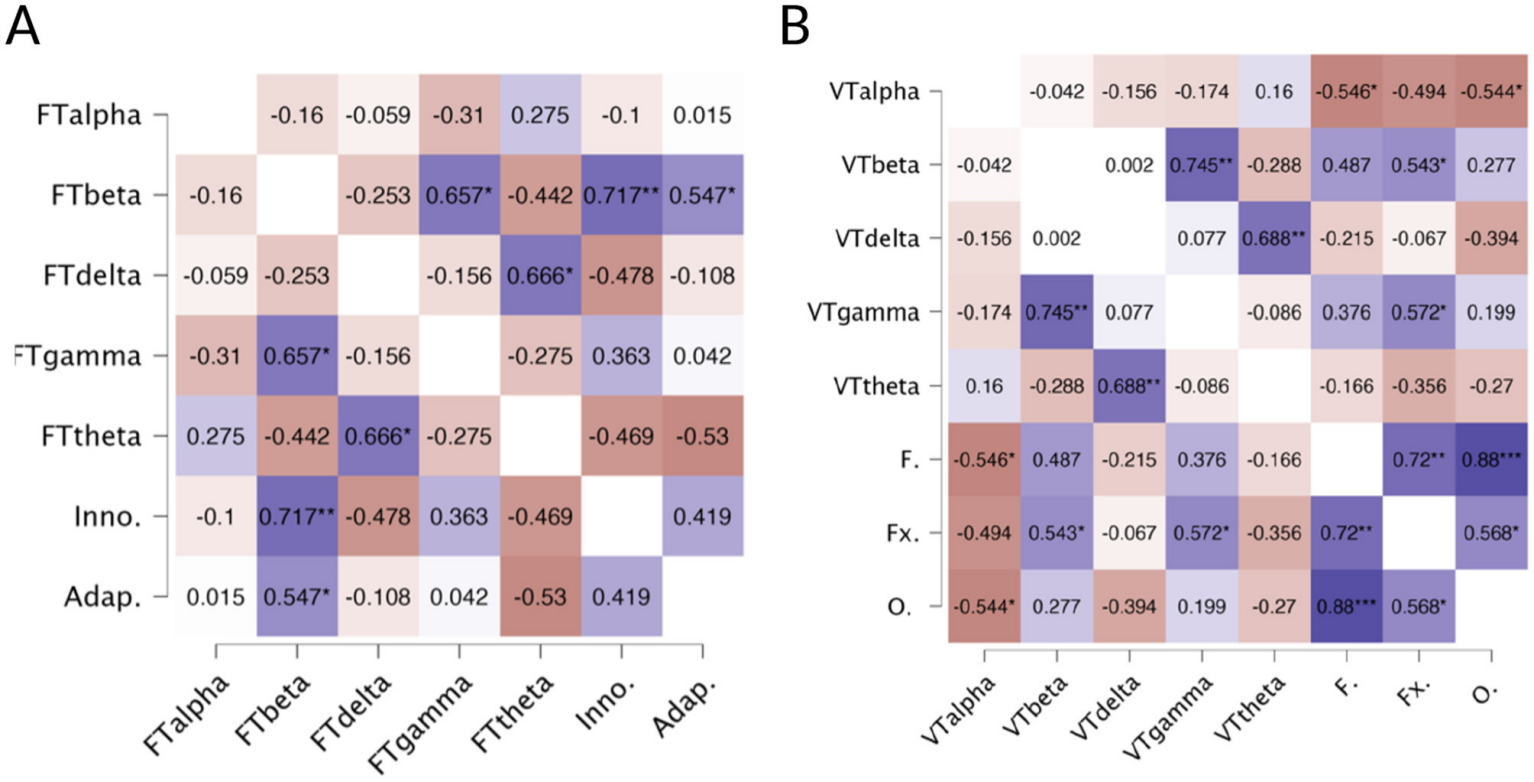
**(A)** Heatmap of correlations between TTCT Figural factors (Innovative and Adaptive) and EEG-based parameters. FTalpha, Figural Test Alpha; FTbeta, Figural Test Beta; FTdelta, Figural Test Delta; FTgamma, Figural Test Gamma; FTtheta, Figural Test Theta; Inno., Innovative Factor; Adap., Adaptive Factor. Blue color indicates positive correlations, and red indicates negative correlations. ^*^p < 0.05, ^**^p < 0.01. **(B)** Heatmap of correlations between TTCT Verbal factors and EEG-based parameters. Note: VTalpha, Verbal Test Alpha; VTbeta, Verbal Test Beta; VTdelta, Verbal Test Delta; VTgamma, Verbal Test Gamma; VTtheta, Verbal Test Theta; F., Fluency; Fx., Flexibility; O., Originality. Blue color indicates positive correlations and red indicates negative correlations (^*^p < 0.05,^**^p < 0.01,^***^p < 0.001).

For the TTCT figural (Figure 3A), results revealed significant positive correlations between the Beta band and both the Innovative factor (*r* = 0.717, *p* < 0.01) and the Adaptive factor (*r* = 0.547, *p* < 0.05). In the verbal task (Figure 3B), a significant positive correlation was found between the Beta band and the Flexibility factor (*r* = 0.543, *p* < 0.05) and between the Gamma band and Flexibility (*r* = 0.572, *p* < 0.05). In contrast, the Alpha band exhibited negative correlations with both Fluency (*r* = −0.546, *p* < 0.05) and Originality (*r* = −0.544, *p* < 0.05).

### 3.3 Coherence analysis

**Figure 4:**
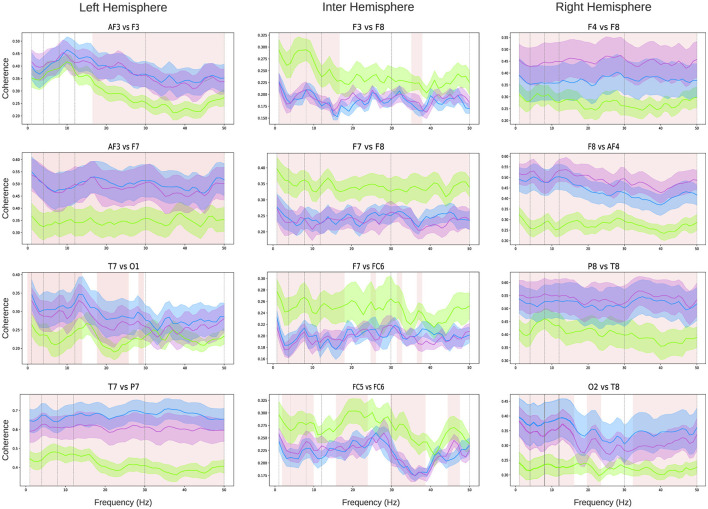
Coherence analysis: intra-hemispheric coherence analysis **(left and right columns)** and inter-hemispheric coherence analysis **(central column)**. Each graph represents the coherence between the channels specified in the title within the frequency range [0–50] Hz. Green lines indicate mean values, and the shaded areas represent the standard deviation across all children: green for the baseline state, violet for the verbal test, and light blue for the figure test. Pink shading highlights the frequency bands where differences between the test conditions and the baseline are statistically significant (the shaded area represents a statistic with *p* < 0.05 using the Kruskal-Wallis test, followed by Dunn's multiple comparisons test).

Coherence analysis was conducted across all pairs of EEG channels. Given the number of comparisons is *N* = 91, Figure 4 highlights the relationships for four pairs intra-hemispheric electrodes on the left and right hemispheres, as well as four pairs of inter-hemispheric connections. The coherence analysis for all channel pairs is presented in Supplementary Figures S2–S6. A clear observation from the intra-hemispheric connectivity analysis is that both the left and right hemispheres exhibit a significant increase (highlighted as shaded pink squares in the figure) in coherence during the figure (violet line) and verbal tasks (blue line) compared to the baseline state (green line). For the left hemisphere (Figure 4 left column), the frontal channel relationship (AF3-F3) shows increased coherence in both the Beta and full Gamma bands. For the AF3-F7 electrode pair, coherence increases across all bands during both tasks. The temporo-occipital relationship (T7-O1) demonstrates an increase in coherence within the Delta, Theta, Alpha, and most of the Beta band. Finally, the temporo-parietal relationship (T7-P7) shows increased coherence across all frequency bands. Similarly, for the right hemisphere (Figure 4 right column), the frontal electrode relationships (F4-F8) and (F8-AF4) display significantly higher coherence during both tasks across all frequency bands. A similar pattern is observed for the parieto-temporal relationship (P8-T8). For the temporal-occipital connection (O2-T8), coherence increases in the Delta, Theta, Alpha, Low-beta, and most of the Gamma bands. In contrast to intra-hemispheric relationships, inter-hemispheric connections (Figure 4 center column) exhibit a general trend of decreased coherence when children perform both tasks. However, this decrease is significant only for electrode pairs in the frontal area (for example F3-F8, F7-F8, F7-FC6, FC5-FC6). For the other electrode pairs, no clear or significant trends are observed (see Supplementary material S2). For a more concise representation of the coherence results across bands, a connected topoplot was generated encompassing all bands (Supplementary Figure S7).

## 4 Discussion

This study aimed to examine the characteristics of the power spectrum and its relationship to behavioral performance, as well as the functional brain connectivity/dysconnectivity within and between hemispheres during the performance of two divergent thinking tasks, including both verbal and figural components, in school-aged children.

First, our results indicated an increase in relative power spectral density (rPSD) during creative task performance compared to the baseline state. This pattern was consistent across all analyzed frequency bands: i.e., theta, alpha, beta and gamma. Our findings align with previous research on brain activity during divergent thinking tasks, reporting increases in the alpha (Benedek, 2018; Fink and Benedek, 2014), Theta (Danko et al., 2003; Jin et al., 2006; Shemyakina et al., 2007), Beta (Danko et al., 2009; Razumnikova, 2007), and Gamma (Bhattacharya and Petsche, 2002; Jung-Beeman et al., 2004) bands. Regarding the delta band, while some studies suggest a decrease in activity during divergent thinking tasks (Boot et al., 2017; Wokke et al., 2019), increases in power have also been observed in specific temporal regions during verbal creativity tasks (Danko et al., 2003). The simultaneous activation of multiple bands aligns with the current perspective on creativity, which is understood as a phenomenon arising from the dynamic interaction between distributed neural networks (Dietrich, 2024), involving the activation of default mode networks during spontaneous idea generation and the engagement of central executive networks during idea elaboration (Pearl, 2024).

Second, the correlation analysis revealed that in the figure task, higher levels of innovation and adaptation were linked to increased activity in the beta band. This suggests that this drawing task may engage processes related to focused attention and sensory processing in children. This finding is consistent with previous research (Razoumnikova, 2000; Stevens Jr and Zabelina, 2020), which reported increased beta activity during creative tasks. In the verbal task, both beta and gamma were positively correlated with flexibility, while alpha showed a negative relationship with fluency and originality in creative responses. These results align with research indicating changes in these bands during creative problem-solving tasks (Vidal et al., 2006) and suggest that creative writing engages processes related to focused attention (beta) and complex processing (gamma). Our findings are also consistent with earlier studies on language production (Luft et al., 2018; ElShafei et al., 2022), that demonstrate a negative correlation between alpha power and behavioral performance (ElShafei et al., 2022; van Ede et al., 2017), suggesting that alpha may enhance task performance by regulating inhibition in areas associated with lexical retrieval (Zioga et al., 2024). Some authors have argued that the dynamics of alpha and beta waves are essential for language comprehension, supporting higher-order processes such as syntactic processing (Meyer, 2017; Zioga et al., 2024). The effects observed in the beta band may indicate an increased demand for retrieving linguistic representations from memory (Hagoort, 2013).

Finally, we observed a significant increase in coherence within each hemisphere during both creative tasks, especially noticeable between frontal-temporal and temporal-occipital regions. These results align with Fink et al. (2009), which identified significant synchronization patterns between frontal and temporal areas during creative tasks. According to Rominger et al. (2022), these patterns may indicate different facets of the creative process; changes in the temporal regions could be related to memory and associative processes, which are fundamental for creative thinking. At the same time, increased coherence in the occipital regions may be linked to internal attention and the suppression of external information, both of which are essential for creative processing. Relatedly, Cruz-Garza et al. (2020) noted that during the preparation phase, there is a flow of connectivity from the frontal areas to the temporal-parietal regions. This flow is associated with the integration of multisensory information. In contrast, during the generation phase, the direction of this flow is reversed, indicating that the integrated information is transformed into tangible creative products. This bidirectional dynamic indicates that the creative process depends not only on the strength of the connections between different brain regions but also on the direction of information flow. Research by Volf and Razumnikova (1999) and Fink and Neubauer (2006) also supports the connection between alpha and theta frequency bands concerning originality and the generation of creative ideas. Overall, the integrated pattern of connectivity indicates that various cognitive processes are coordinated in a complex manner during creative task performance, with different brain regions collaborating to facilitate both the generation and processing of creative and innovative ideas, revealing the diverse facets of the creative process. According to Dietrich and Kanso (2010) (see also Ceauşu, 2024), creativity engages an extensive network of brain structures across both hemispheres. Additionally, our results indicate a decrease in interhemispheric coherence during both tasks, with a more significant effect observed in the frontal region. These findings are consistent with Pearl (2024), which found that creative processes involve the deactivation of both the dorsolateral and lateral orbital prefrontal regions, while the medial prefrontal cortex remains active. The reduction in frontal interhemispheric coherence may represent a neural mechanism that facilitates creativity by temporarily reducing executive control. Pearl (2024) suggests that a more relaxed and less constrained mental state, characterized by reduced cognitive control, may enhance creativity, while excessive executive control could hinder it. The decrease in frontal interhemispheric coherence may indicate a functional adaptation in the brain, enabling greater flexibility in information processing. This flexibility could facilitate the generation and exploration of creative ideas.

Before discussing the implications of the study, we must recognize some limitations. First, our sample consisted of a small group of middle-class children. Besides, while previous research has validated the Emotiv Epoc+'s ability to capture brain activity patterns (Bobrov et al., 2011; Amjad et al., 2019), its spatial resolution is not as high as that of other higher-density systems. Future research would benefit from examining the relationship between the specific facets of creativity and their associated neural patterns, using both divergent and convergent thinking tasks to gain a clearer understanding of the neural mechanisms underlying creativity in children. Additionally, future studies could explore how different cognitive processes interact during creative tasks, which may help clarify the observed correlations between brain activity and behavioral performance. Finally, a promising direction for future research is developing a normative EEG database focused on creativity. This database would include measurements such as relative Power Spectral Density (rPSD) and coherence across the different frequency bands during various creative tasks. While results can vary significantly due to factors like age, sex, educational level, and the type of creative task, a well-constructed database could help identify general patterns and explore how these characteristics affect brain activity. The major challenges include controlling for individual variables and ensuring a representative sample. In the long term, such a database could enhance our understanding of the brain mechanisms underlying creativity and have clinical applications for conditions such as autism, ADHD, and age-related disorders.

The present findings have important theoretical and practical implications. Theoretically, our results underline the complexity of creativity by differentiating between overall brain activation during creative tasks and specific associations between EEG-based parameters and creativity performance. While all frequency bands were activated during both tasks, our correlations indicate that only certain neurophysiological processes are linked to specific aspects of creative performance in children. Despite the small sample size, these results provide a basis for further investigation into how specific brain dynamics contribute to creativity. From a practical perspective, the overall increase in brain activity suggests that creativity engages multiple cognitive processes, including attention, flexibility, and the ability to form conceptual connections (Mateos et al., 2022). This process reflects a neural orchestration in which various regions of the brain work together.

## 5 Conclusions

We observed a significant increase in brain activity (rPSD) across multiple frequency bands, indicating that the creativity process involves the engagement of different mental processes through its distinct phases. Correlations between EEG-based parameters and behavioral performance yielded interesting results. In the figural task, greater levels of innovation and adaptation were linked to increased activity in the beta band. For the verbal task, flexibility showed a positive correlation with activity in both the beta and gamma bands. Additionally, fluency and originality were negatively related to the alpha band. We also observed a reorganization in brain communication, characterized by increased connectivity within the hemispheres and reduced connectivity between them in the frontal area. This suggests a balance between information integration and mental flexibility. These findings deepen our understanding of childhood creativity as a complex process that involves the coordinated interaction of various brain regions.

## Data Availability

The raw data supporting the conclusions of this article will be made available by the authors, without undue reservation.

## References

[B1] Ahad M. T. Hartog T. Alhashim A. G. Marshall M. Siddique Z. (2023). Electroencephalogram experimentation to understand creativity of mechanical engineering students. ASME Open J. Eng. 2:21005. 10.1115/1.4056473

[B2] Amabile T. M. (1983). The social psychology of creativity: A componential conceptualization. J. Pers. Soc. Psychol. 45, 357. 10.1037/0022-3514.45.2.357

[B3] Amjad I. Toor H. Niazi I. K. Afzal H. Jochumsen M. Shafique M.. (2019). Therapeutic effects of aerobic exercise on EEG parameters and higher cognitive functions in mild cognitive impairment patients. Int. J. Neurosci. 129, 551–562. 10.1080/00207454.2018.1551894 30929591

[B4] Arden R. Chavez R. S. Grazioplene R. Jung R. E. (2010). Neuroimaging creativity: a psychometric view. Behav. Brain Res. 214, 143–156. 10.1016/j.bbr.2010.05.015 20488210

[B5] Bartoli E. Devara E. Dang H. Q. Rabinovich R. Mathura R. K. Anand A.. (2024). Default mode network electrophysiological dynamics and causal role in creative thinking. Brain 147, 3409–3425. 10.1093/brain/awae199 38889248 PMC11449134

[B6] Bazanova O. Aftanas L. (2008). Individual measures of electroencephalogram alpha activity and non-verbal creativity. Neurosci. Behav. Physiol. 38, 227–235. 10.1007/s11055-008-0034-y 18264769

[B7] Beaty R. E. Benedek M. Barry Kaufman S. Silvia P. J. (2015). Default and executive network coupling supports creative idea production. Sci. Rep. 5:10964. 10.1038/srep10964 26084037 PMC4472024

[B8] Benedek M. (2018). The Neuroscience of Creative Idea Generation. Cham: Springer International Publishing, 31–48. 10.1007/978-3-319-76054-4_2

[B9] Benedek M. Bergner S. Könen T. Fink A. Neubauer A. C. (2011). EEG alpha synchronization is related to top-down processing in convergent and divergent thinking. Neuropsychologia 49, 3505–3511. 10.1016/j.neuropsychologia.2011.09.004 21925520 PMC3198250

[B10] Benedek M. Franz F. Heene M. Neubauer A. C. (2012a). Differential effects of cognitive inhibition and intelligence on creativity. Pers. Individ. Dif. 53, 480–485. 10.1016/j.paid.2012.04.014 22945970 PMC3387381

[B11] Benedek M. Könen T. Neubauer A. C. (2012b). Associative abilities underlying creativity. Psychol. Aesthet. Creat. Arts 6:273. 10.1037/a0027059

[B12] Bhattacharya J. Petsche H. (2002). Shadows of artistry: cortical synchrony during perception and imagery of visual art. Cogn. Brain Res. 13, 179–186. 10.1016/S0926-6410(01)00110-0 11958960

[B13] Bhattacharya J. Petsche H. (2005). Drawing on mind's canvas: differences in cortical integration patterns between artists and non-artists. Hum. Brain Mapp. 26, 1–14. 10.1002/hbm.20104 15852480 PMC6871726

[B14] Bobrov P. Frolov A. Cantor C. Fedulova I. Bakhnyan M. Zhavoronkov A. (2011). Brain-computer interface based on generation of visual images. PLoS ONE 6:e20674. 10.1371/journal.pone.0020674 21695206 PMC3112189

[B15] Boden M. A. (1998). Creativity and artificial intelligence. Artif. Intell. 103, 347–356. 10.1016/S0004-3702(98)00055-1

[B16] Boot N. Baas M. Mühlfeld E. de Dreu C. K. van Gaal S. (2017). Widespread neural oscillations in the delta band dissociate rule convergence from rule divergence during creative idea generation. Neuropsychologia 104, 8–17. 10.1016/j.neuropsychologia.2017.07.033 28774832

[B17] Cavanagh J. F. Frank M. J. (2014). Frontal theta as a mechanism for cognitive control. Trends Cogn. Sci. 18, 414–421. 10.1016/j.tics.2014.04.012 24835663 PMC4112145

[B18] Ceauşu F. (2024). Brain and creativity. Rev. Artis. Educ. 28, 285–297. 10.35218/rae-2024-0034

[B19] Cruz-Garza J. G. Sujatha Ravindran A. Kopteva A. E. Rivera Garza C. Contreras-Vidal J. L. (2020). Characterization of the stages of creative writing with mobile EEG using generalized partial directed coherence. Front. Hum. Neurosci. 14:577651. 10.3389/fnhum.2020.577651 33424562 PMC7793781

[B20] Csikszentmihalyi M. (1988). The Flow Experience and its Significance for Human Psychology. Cambridge: Cambridge University Press, 15–35. 10.1017/CBO9780511621956.002

[B21] Danko S. Shemyakina N. Nagornova Z. V. Starchenko M. (2009). Comparison of the effects of the subjective complexity and verbal creativity on EEG spectral power parameters. Hum. Physiol. 35, 381–383. 10.1134/S0362119709030153 19534413

[B22] Danko S. G. Starchenko M. G. Bechtereva N. P. (2003). EEG local and spatial synchronization during a test on the insight strategy of solving creative verbal tasks. Hum. Physiol. 29, 502–504. 10.1023/A:1024950028210 13677209

[B23] Dietrich A. (2024). Where in the brain is creativity? The fallacy of a creativity faculty in the brain. Front. Psychol. 15:1373299. 10.3389/fpsyg.2024.1373299 38746914 PMC11091241

[B24] Dietrich A. Kanso R. (2010). A review of EEG, erp, and neuroimaging studies of creativity and insight. Psychol. Bull. 136:822. 10.1037/a0019749 20804237

[B25] ElShafei H. A. Zhou Y. J. Haegens S. (2022). Shaping information processing: the role of oscillatory dynamics in a working memory task. Eneuro 9:ENEURO.0489–21.2022. 10.1523/ENEURO.0489-21.2022 35977824 PMC9480873

[B26] Emotiv (2021). Emotiv epoc+. Available on: https://www.emotiv.com/epoc/ (accessed September 8, 2021).

[B27] Eymann V. Lachmann T. Beck A.-K. Czernochowski D. (2024). EEG oscillatory evidence for the temporal dynamics of divergent and convergent thinking in the verbal knowledge domain. Intelligence 104:101828. 10.1016/j.intell.2024.101828

[B28] Fink A. Benedek M. (2014). EEG alpha power and creative ideation. Neurosci. Biobehav. Rev. 44, 111–123. 10.1016/j.neubiorev.2012.12.002 23246442 PMC4020761

[B29] Fink A. Grabner R. H. Benedek M. Reishofer G. Hauswirth V. Fally M.. (2009). The creative brain: investigation of brain activity during creative problem solving by means of EEG and fmri. Hum. Brain Mapp. 30, 734–748. 10.1002/hbm.20538 18266217 PMC6871103

[B30] Fink A. Neubauer A. C. (2006). EEG alpha oscillations during the performance of verbal creativity tasks: differential effects of sex and verbal intelligence. Int. J. Psychophysiol. 62, 46–53. 10.1016/j.ijpsycho.2006.01.001 16503062

[B31] Gra nana N. Richaudeau A. Gorriti C. R. O'Flaherty M. Scotti M. E. Sixto L.. (2011). Evaluación de déficit de atención con hiperactividad: la escala snap iv adaptada a la argentina. Rev. Panamericana Salud Pública 29, 344–349. 10.1590/S1020-49892011000500007 21709939

[B32] Grabner R. H. Fink A. Neubauer A. C. (2007). Brain correlates of self-rated originality of ideas: evidence from event-related power and phase-locking changes in the EEG. Behav. Neurosci. 121:224. 10.1037/0735-7044.121.1.224 17324067

[B33] Gramfort A. Luessi M. Larson E. Engemann D. A. Strohmeier D. Brodbeck C.. (2013). MEG and EEG data analysis with mne-python. Front. Neuroinform. 7:267. 10.3389/fnins.2013.00267 24431986 PMC3872725

[B34] Guilford J. P. (1956). The structure of intellect. Psychol. Bull. 53:267. 10.1037/h0040755 13336196

[B35] Hagoort P. (2013). Muc (memory, unification, control) and beyond. Front. Psychol. 4:416. 10.3389/fpsyg.2013.00416 23874313 PMC3709422

[B36] Jauk E. Benedek M. Neubauer A. C. (2012). Tackling creativity at its roots: evidence for different patterns of EEG alpha activity related to convergent and divergent modes of task processing. Int. J. Psychophysiol. 84, 219–225. 10.1016/j.ijpsycho.2012.02.012 22390860 PMC3343259

[B37] Jin S.-H. Kwon Y.-J. Jeong J.-S. Kwon S.-W. Shin D.-H. (2006). Differences in brain information transmission between gifted and normal children during scientific hypothesis generation. Brain Cogn. 62, 191–197. 10.1016/j.bandc.2006.05.001 16766109

[B38] Jung-Beeman M. Bowden E. M. Haberman J. Frymiare J. L. Arambel-Liu S. Greenblatt R.. (2004). Neural activity when people solve verbal problems with insight. PLoS Biol. 2:e97. 10.1371/journal.pbio.0020097 15094802 PMC387268

[B39] Krumm G. Aranguren M. Arán Filippetti V. Lemos V. (2016a). Factor structure of the torrance tests of creative thinking verbal form b in a spanish-speaking population. J. Creat. Behav. 50, 150–164. 10.1002/jocb.76

[B40] Krumm G. Filipppetti V. A. Lemos V. Koval J. Balabanian C. (2016b). Construct validity and factorial invariance across sex of the torrance test of creative thinking-figural form a in spanish-speaking children. Think. Skills Creativ. 22, 180–189. 10.1016/j.tsc.2016.10.003

[B41] Luft C. D. B. Zioga I. Thompson N. M. Banissy M. J. Bhattacharya J. (2018). Right temporal alpha oscillations as a neural mechanism for inhibiting obvious associations. Proc. Nat. Acad. Sci. 115, E12144–E12152. 10.1073/pnas.1811465115 30541890 PMC6310824

[B42] Mateos D. M. Krumm G. Arán Filippetti V. Gutierrez M. (2022). Power spectrum and connectivity analysis in EEG recording during attention and creativity performance in children. NeuroSci 3, 347–365. 10.3390/neurosci3020025

[B43] Mednick S. (1962). The associative basis of the creative process. Psychol. Rev. 69:220. 10.1037/h0048850 14472013

[B44] Meyer L. (2017). The neural oscillations of speech processing and language comprehension: state of the art and emerging mechanisms. Eur. J. Neurosci. 48, 2609–2621. 10.1111/ejn.13748 29055058

[B45] Pearl P. L. (2024). The neurology of creativity: 2023 hower lecture. Ann. Child Neurol. Soc. 2, 6–14. 10.1002/cns3.20067 PMC13359223 42563848

[B46] Pfenninger K. H. Shubik V. (2001). “Insights into the foundation of creativity: a synthesis,” in Originals Creativity, 213–236.

[B47] Pidgeon L. M. Grealy M. Duffy A. H. Hay L. McTeague C. Vuletic T.. (2016). Functional neuroimaging of visual creativity: A systematic review and meta-analysis. Brain Behav. 6:e00540. 10.1002/brb3.540 27781148 PMC5064346

[B48] Raven J. C. C. J. H. Raven J. (2008). Test de matrices progresivas. Escalas coloreada, general y avanzada. Manual. Paidós.

[B49] Razoumnikova O. M. (2000). Functional organization of different brain areas during convergent and divergent thinking: an EEG investigation. Cogn. Brain Res. 10, 11–18. 10.1016/S0926-6410(00)00017-3 10978688

[B50] Razumnikova O. Volf N. Tarasova I. (2009). Strategy and results: Sex differences in electrographic correlates of verbal and figural creativity. Hum. Physiol. 35, 285–294. 10.1134/S0362119709030049 19534402

[B51] Razumnikova O. M. (2007). Creativity related cortex activity in the remote associates task. Brain Res. Bull. 73, 96–102. 10.1016/j.brainresbull.2007.02.008 17499642

[B52] Rominger C. Benedek M. Lebuda I. Perchtold-Stefan C. M. Schwerdtfeger A. R. Papousek I.. (2022). Functional brain activation patterns of creative metacognitive monitoring. Neuropsychologia 177:108416. 10.1016/j.neuropsychologia.2022.108416 36343705

[B53] Rominger C. Papousek I. Perchtold C. M. Benedek M. Weiss E. M. Schwerdtfeger A.. (2019). Creativity is associated with a characteristic u-shaped function of alpha power changes accompanied by an early increase in functional coupling. Cogn. Affect. Behav. Neurosci. 19, 1012–1021. 10.3758/s13415-019-00699-y 30756348 PMC6711878

[B54] Shemyakina N. Danko S. Nagornova Z. V. Starchenko M. Bechtereva N. (2007). Changes in the power and coherence spectra of the EEG rhythmic components during solution of a verbal creative task of overcoming a stereotype. Hum. Physiol. 33, 524–530. 10.1134/S0362119707050027 18038659

[B55] Simonton D. (1988). Scientific Genius: A Psychology of Science. Cambridge: Cambridge University Press.

[B56] Sternberg R. J. Lubart T. I. (1993). Investing in creativity. Psychol. Inq. 4, 229–232. 10.1207/s15327965pli0403_16

[B57] Stevens Jr C. E. Zabelina D. L. (2019). Creativity comes in waves: an EEG-focused exploration of the creative brain. Curr. Opin. Behav. Sci. 27, 154–162. 10.1016/j.cobeha.2019.02.003

[B58] Stevens Jr C. E. Zabelina D. L. (2020). Classifying creativity: applying machine learning techniques to divergent thinking EEG data. Neuroimage 219:116990. 10.1016/j.neuroimage.2020.116990 32474083

[B59] Stoica P. Moses R. L. (2005). Spectral Analysis of Signals. Upper Saddle River, NJ: Pearson Prentice Hall.

[B60] Sun C. Zhou Z. (2024). Electroencephalography (EEG) evidence for the psychological processes of humor generation: A comparison perspective on humor and creativity. Behav. Sci. 14:290. 10.3390/bs14040290 38667085 PMC11047550

[B61] Torrance E. (1990). Tests of Creative Thinking: Streamlined Scoring Guide-Figural and Verbal a and b. Bensenville (IL): Scholastic Testing Service.

[B62] Torrance E. Ball O. Safter H. (1992). Torrance Test of Creative Thinking. Streamlined Scoring Guide Figural a and b. Bensenville, Illinois: Scholastic testing service. Inc. 4p.

[B63] van Ede F. Jensen O. Maris E. (2017). Supramodal theta, gamma, and sustained fields predict modality-specific modulations of alpha and beta oscillations during visual and tactile working memory. J. Cogn. Neurosci. 29, 1455–1472. 10.1162/jocn_a_01129 28358658

[B64] Vidal J. R. Chaumon M. O'Regan J. K. Tallon-Baudry C. (2006). Visual grouping and the focusing of attention induce gamma-band oscillations at different frequencies in human magnetoencephalogram signals. J. Cogn. Neurosci. 18, 1850–1862. 10.1162/jocn.2006.18.11.1850 17069476

[B65] Volf N. V. Razumnikova O. M. (1999). Sex differences in EEG coherence during a verbal memory task in normal adults. Int. J. Psychophysiol. 34, 113–122. 10.1016/S0167-8760(99)00067-7 10576396

[B66] Wokke M. E. Padding L. Ridderinkhof K. (2019). Creative brains show reduced mid frontal theta. bioRxiv, 370494. 10.1101/370494

[B67] Zioga I. Zhou Y. J. Weissbart H. Martin A. E. Haegens S. (2024). Alpha and beta oscillations differentially support word production in a rule-switching task. Eneuro 11:ENEURO.0312–23.2024. 10.1523/ENEURO.0312-23.2024 38490743 PMC10988358

