# Supplementary material for: Exploring the neural basis of creativity: EEG analysis of power spectrum and functional connectivity during creative tasks in school-aged children
Source: Front Comput Neurosci. 2025 Mar 12;19:1548620. doi: 10.3389/fncom.2025.1548620 (PMC11937046; doi:10.3389/fncom.2025.1548620)
Supplement: Supplementary file 1 [file Data_Sheet_1.pdf]

Supplementary Material

1 PARTICIPANT INFORMATION

Basic and Anthropometric Data	
**Characteristics**	**Value**
Age in years (M ± SD)	10.33 ± 1.11
Gender (% girls)	46.7
Birth weight	3.37 ± 0.41 kg
Gestational age (<37 weeks)	100% full-term
Hand dominance	100% right-handed
**Family and Educational Environment**	
**Factor**	**Level/Percentage**
Father's educational level	3.26 ± 1.22
Mother's educational level	3.46 ± 0.92
Exclusive breastfeeding	73.3%
Mixed breastfeeding	26.7%
**Complementary Activities and Training**	
**Activity**	**Participation/Duration**
Soccer/Volleyball	46.6%
Skating/Dance	33.3%
Choir and Sports	20.0%
Years in activities	4.00 ± 1.06
Foreign language study	66.7%
Years studying foreign language	2.26 ± 2.52
**Control Measures**	
**Test**	**Score**
Raven (percentile)	55.53 ± 30.55
SNAP-IV Inattention	0.60 ± 0.67
SNAP-IV Hyperactivity–impulsivity	0.60 ± 0.65
SNAP-IV Combined ADHD	1.20 ± 0.86
**Creativity Measures**	
**Test**	**Score**
TTCT Figural, task 2	10.80 ± 3.91
TTCT Verbal, task 5	8.37 ± 2.67

Table S1. Demographic characteristics of the sample and descriptive statistics of measures. Values are expressed as Mean ± Standard Deviation or percentages. Note: Educational level is measured on a 5-point scale (1=Primary, 2=Secondary, 3=Tertiary non-university, 4=University degree, 5=Postgraduate).

2 POWER SPECTRUM P-VALUES

3 COHERENCE ANALYSIS ALL ELECTRODES PAIRS

4 CONNECTED TOPOPLOT COHERENCE ANALYSIS

To present the coherence correlation results in a more compact manner, the following approach was applied. The mean coherence values depicted in the figures were calculated for each band, yielding, for each band, subject, and condition, a symmetric 14×14 coherence matrix. A Kruskal-Wallis statistical test was performed on each matrix entry, comparing the baseline condition with the figure/verbal tasks. For entries where the test statistic was significant ($p < 0.01$), a delta coherence was computed as the difference

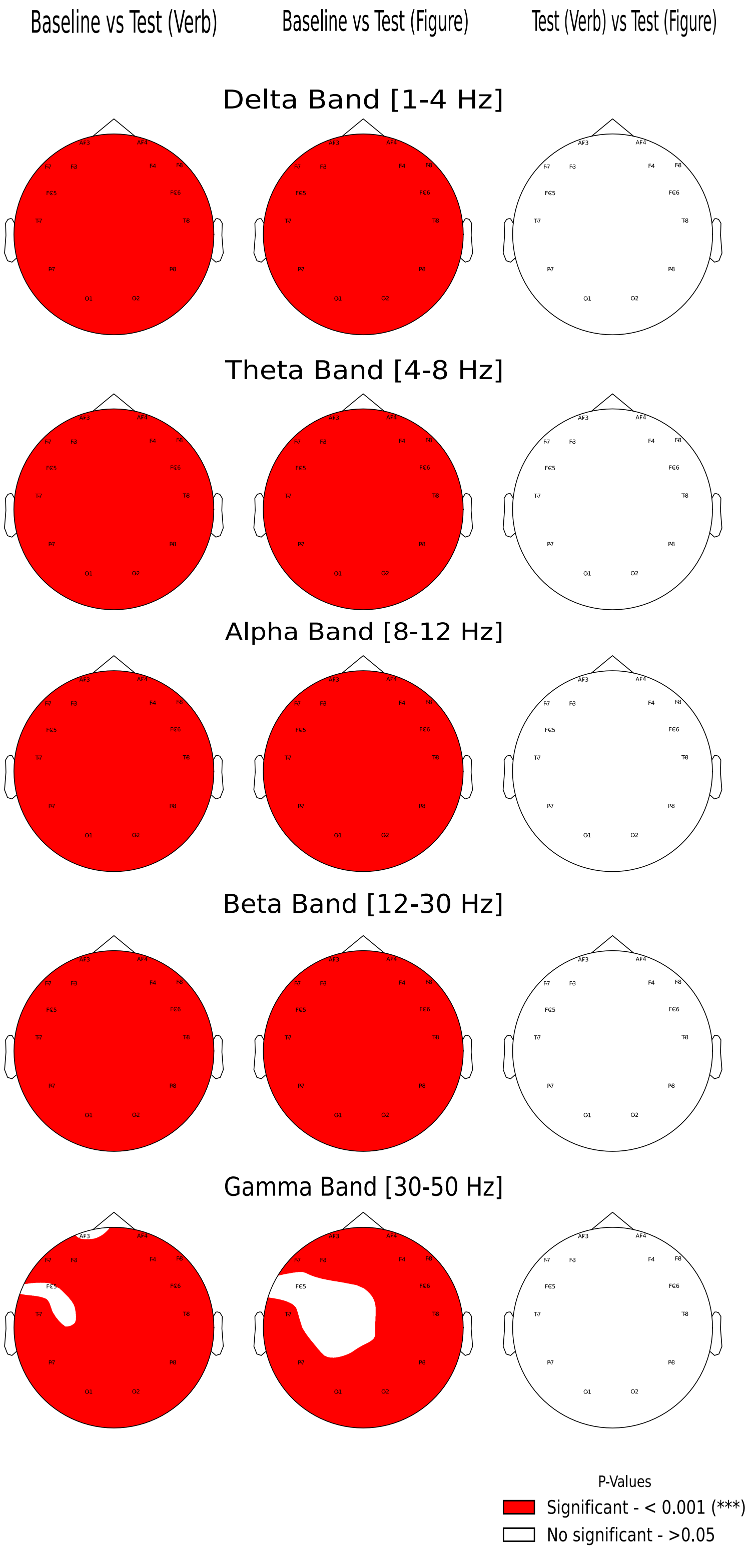

Figure S1. Distribution of p-values comparing the power spectrum values across different frequency bands for various groups: Baseline vs. Verbal Test, Baseline vs. Figure Test, and Verbal Test vs. Figure Test. Statistical comparisons were performed using the Kruskal-Wallis test, followed by Dunn's multiple comparisons test to identify significant differences.

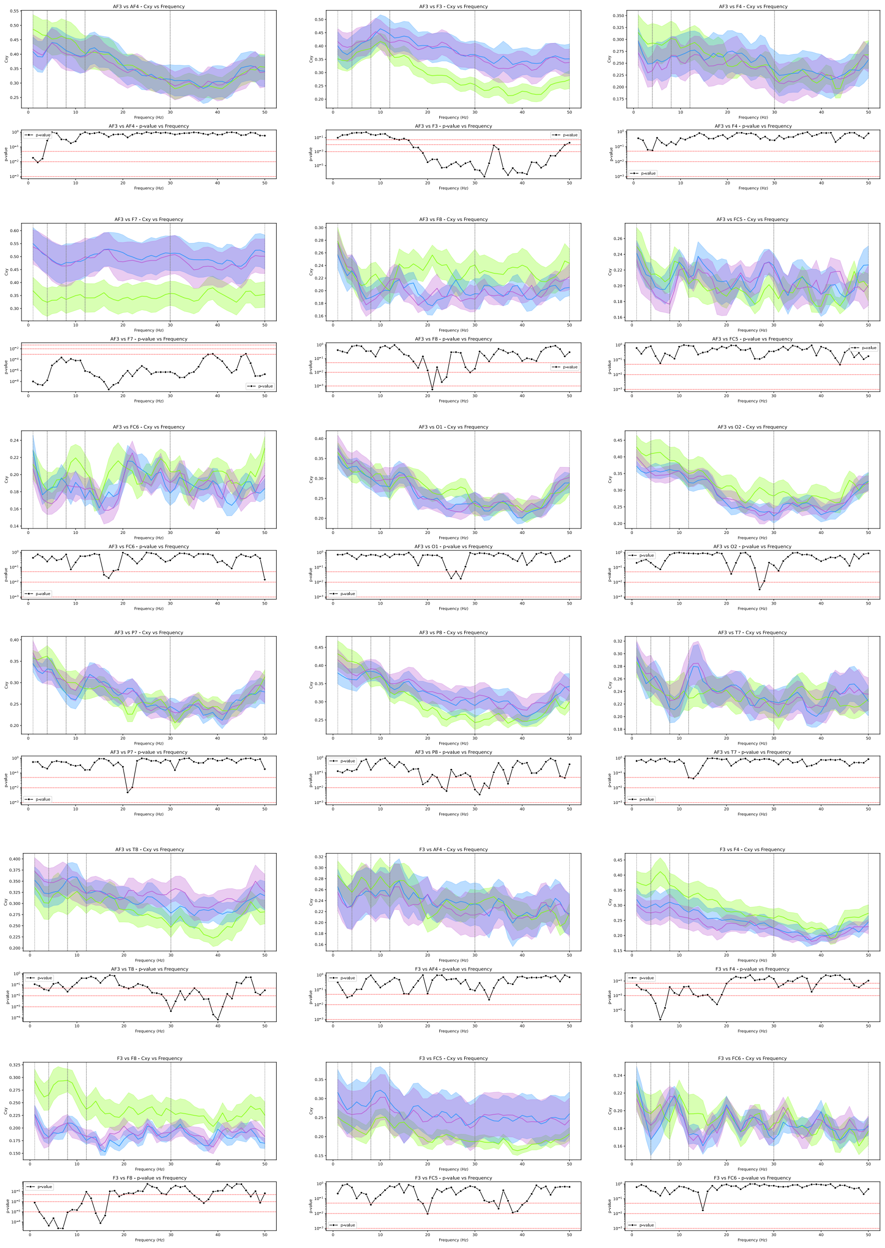

Figure S2. Coherence Analysis: The top graph depicts coherence between the specified channels across the frequency range [0-50 Hz]. The green lines represent mean values, while the shaded regions correspond to the standard deviation across all children: green for the baseline state, violet for the verbal test, and light blue for the figure test. The bottom graph illustrates the p-values for each frequency band analysis. Statistical comparisons were conducted using the Kruskal-Wallis test, followed by Dunn's multiple comparisons test to identify significant differences.

between the figure/verbal tasks and the baseline. This yielded a new difference matrix (figure/verbal – baseline), which was then visualized using a connected topoplot.

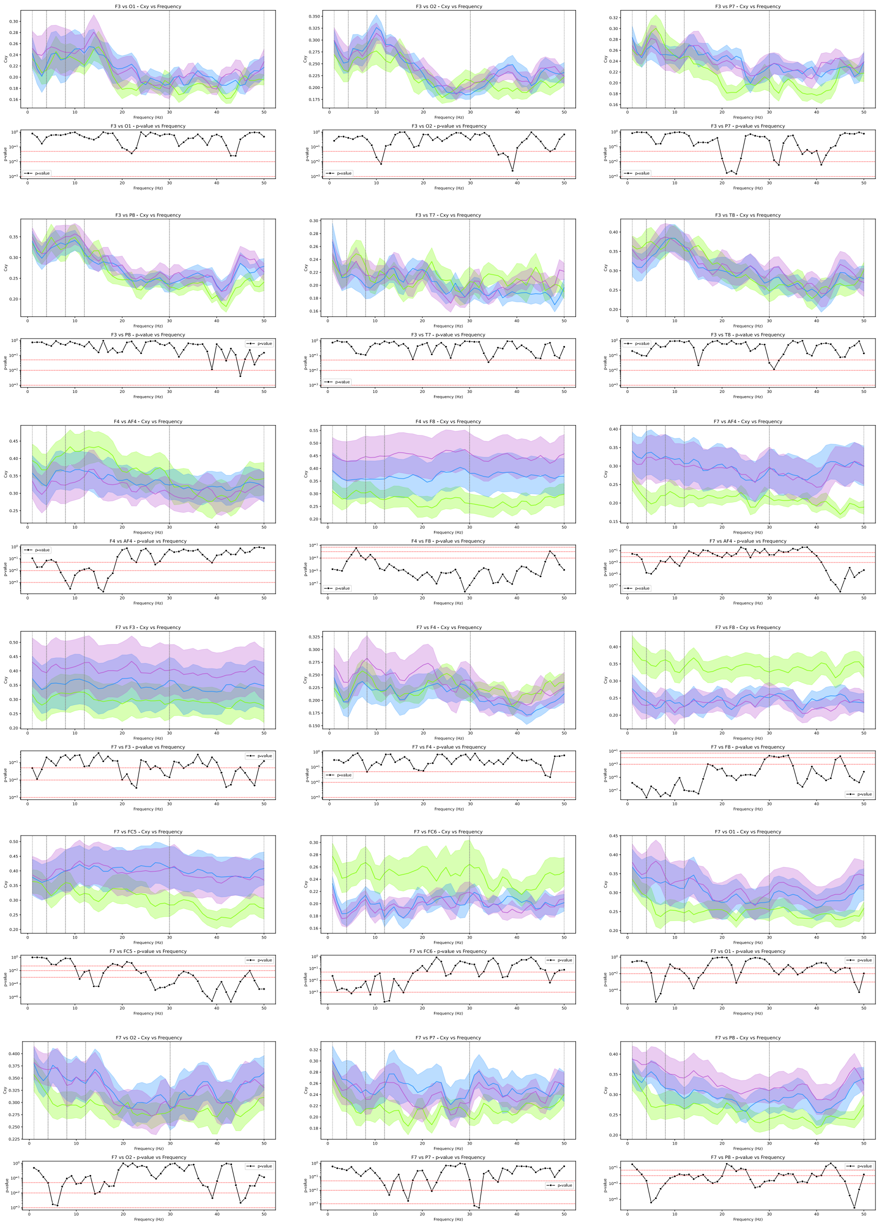

Figure S3. Coherence Analysis: The top graph depicts coherence between the specified channels across the frequency range [0-50 Hz]. The green lines represent mean values, while the shaded regions correspond to the standard deviation across all children: green for the baseline state, violet for the verbal test, and light blue for the figure test. The bottom graph illustrates the p-values for each frequency band analysis. Statistical comparisons were conducted using the Kruskal-Wallis test, followed by Dunn's multiple comparisons test to identify significant differences.

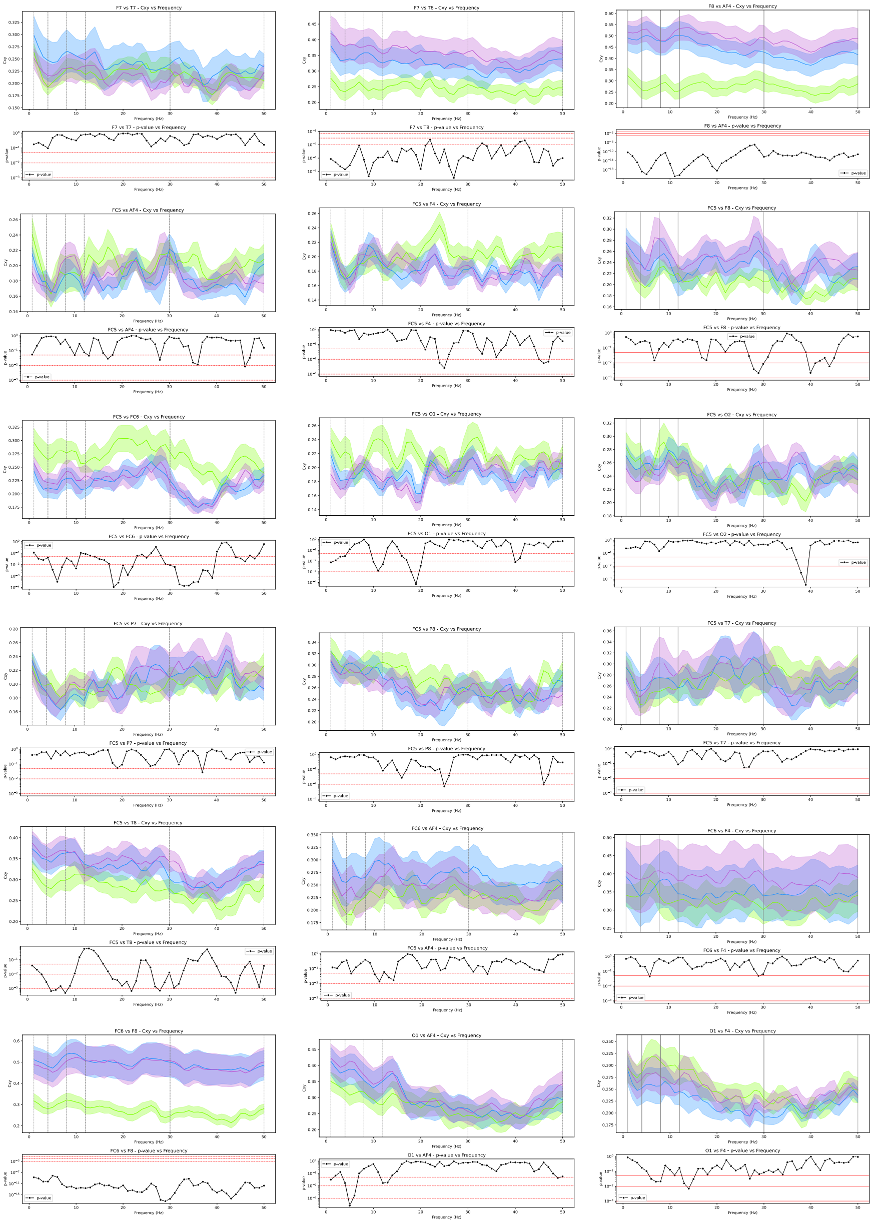

Figure S4. Coherence Analysis: The top graph depicts coherence between the specified channels across the frequency range [0-50 Hz]. The green lines represent mean values, while the shaded regions correspond to the standard deviation across all children: green for the baseline state, violet for the verbal test, and light blue for the figure test. The bottom graph illustrates the p-values for each frequency band analysis. Statistical comparisons were conducted using the Kruskal-Wallis test, followed by Dunn's multiple comparisons test to identify significant differences.

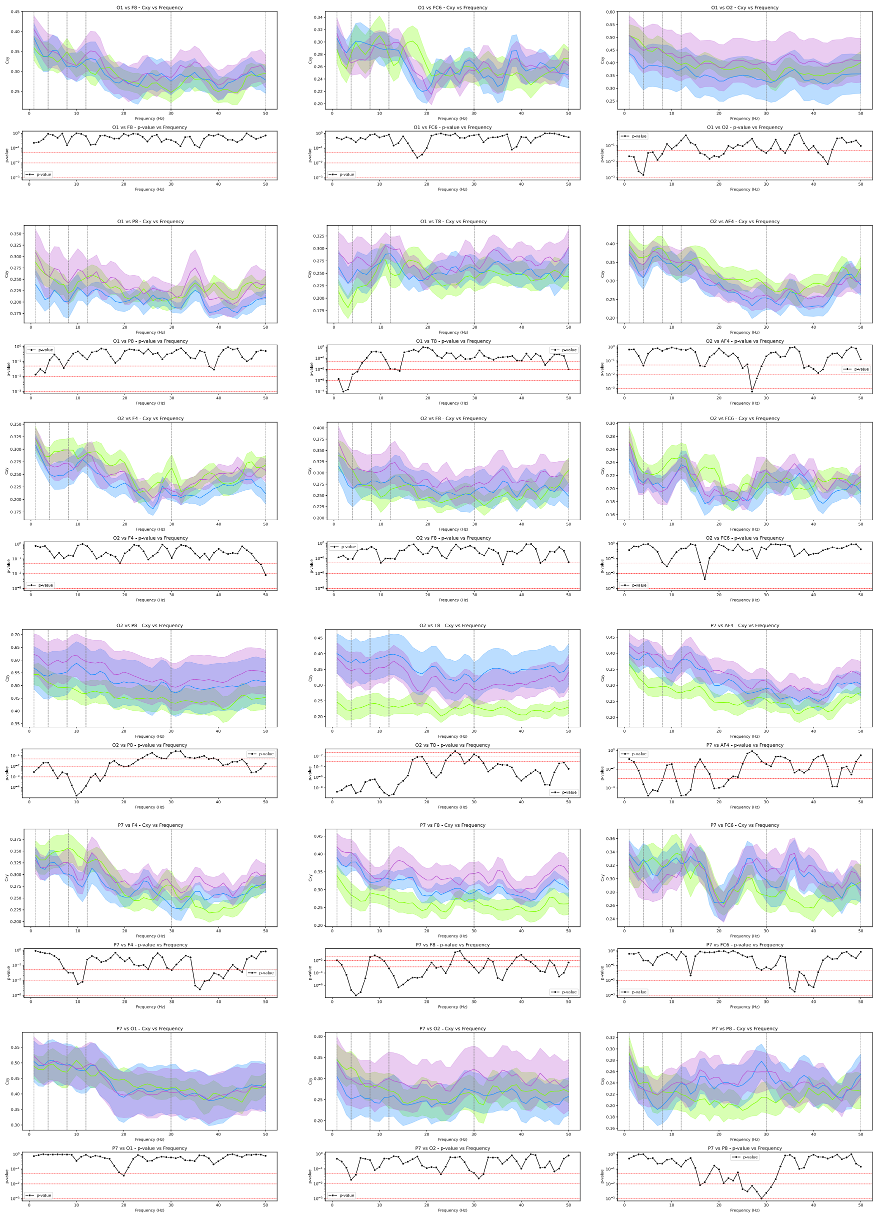

Figure S5. Coherence Analysis: The top graph depicts coherence between the specified channels across the frequency range [0-50 Hz]. The green lines represent mean values, while the shaded regions correspond to the standard deviation across all children: green for the baseline state, violet for the verbal test, and light blue for the figure test. The bottom graph illustrates the p-values for each frequency band analysis. Statistical comparisons were conducted using the Kruskal-Wallis test, followed by Dunn's multiple comparisons test to identify significant differences.

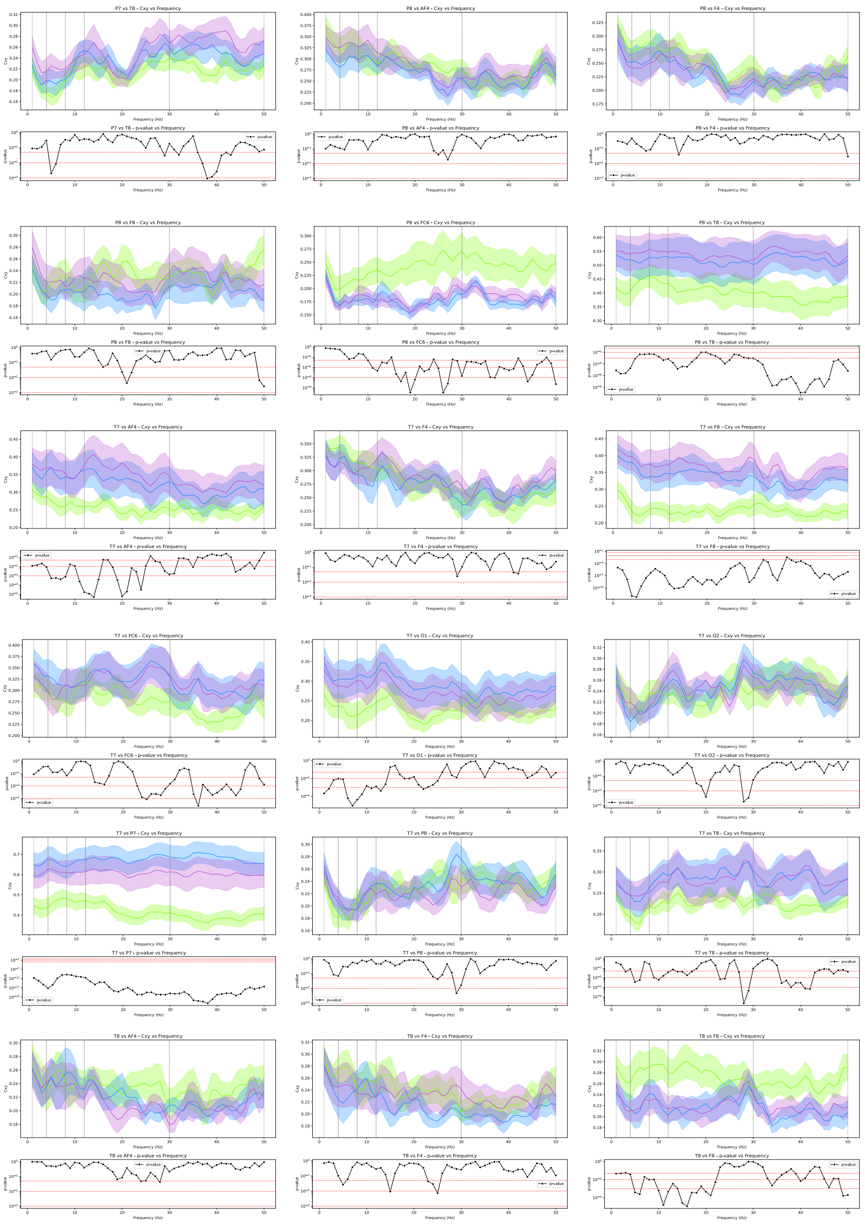

Figure S6. Coherence Analysis: The top graph depicts coherence between the specified channels across the frequency range [0-50 Hz]. The green lines represent mean values, while the shaded regions correspond to the standard deviation across all children: green for the baseline state, violet for the verbal test, and light blue for the figure test. The bottom graph illustrates the p-values for each frequency band analysis. Statistical comparisons were conducted using the Kruskal-Wallis test, followed by Dunn's multiple comparisons test to identify significant differences.

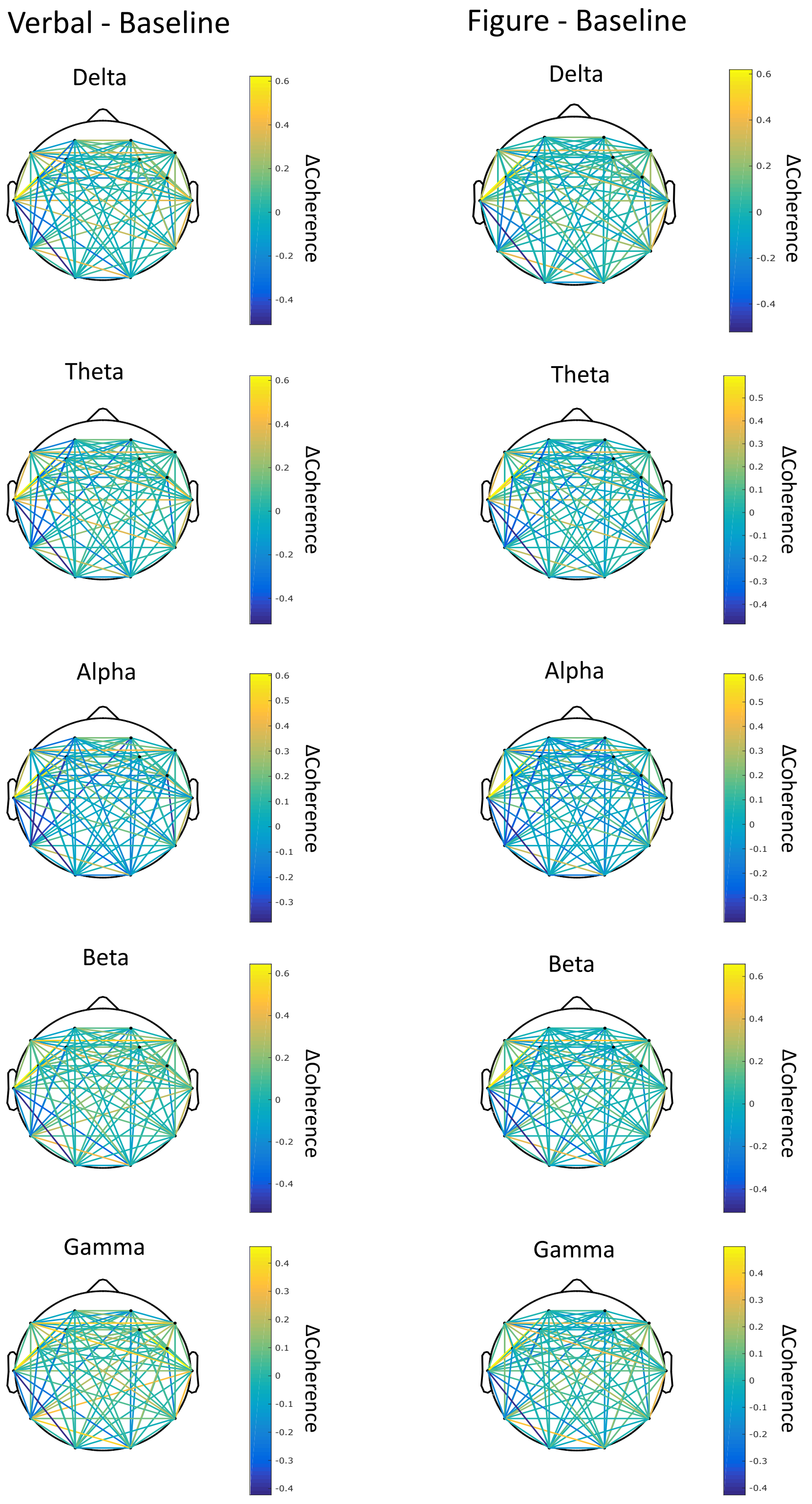

Figure S7. Topoplot Coherence Analysis: Analysis of coherence differences between the verbal task (left column) and figure task (right column) compared to baseline across the analyzed frequency bands. Only connections that were statistically significant between groups (Kruskal-Wallis test, $p < 0.01$) are displayed. Positive values (green to yellow) indicate higher coherence during the task compared to baseline, while negative values (green to blue) indicate the opposite.
